# Supplementary material for: An advanced enrichment method for rare somatic retroelement insertions sequencing
Source: Mob DNA. 2018 Oct 31;9:31. doi: 10.1186/s13100-018-0136-1 (PMC6208084; doi:10.1186/s13100-018-0136-1)
Supplement: Supplementary file 1 — This file contains a table with sequences of oligonucleotide primers used in this study. (PDF 70 kb) [file 13100_2018_136_MOESM1_ESM.pdf]

Oligonucleotides used in this study

AluYa5 flanking fragments library preparation	
St19BH	TGTAGCGTGAAGACGACAGAAAGGGCGTGCTGCGGBBBBHHHHAGGGCGGT
St20BH	ACCGCCCT
AY107 Rev	TCACCGTTTTAGCCGGGA
Na21	TGTAGCGTGAAGACGACAGAA
AY16-6	GCCACCGCGCC
St19okor	GCGTGCTGCGG
Re-amplification after normalization	
AY16-ind301	CAACTGCCACCGCGCC
AY16-ind302	CTGTGGCCACCGCGCC
AY16-ind304	GGAACGCCACCGCGCC
Spike-in preparation	
DR240 For1	GCCACCGCGCCCGGCCTTGTAGTGTGGAATGCAGGTAG
DR240 Rev	CTGAGGCAGCGATGATGG
DR259 For1	GCCACCGCGCCCGGCCGTTGTTTTGTTGGCTGATATTG
DR259 Rev	CTTGCTGGTGAGAAGTTATTTG
DR418 For1	GCCACCGCGCCCGGCCGCAACTCCTTATTCCGCCTG
DR418 Rev	CTCGCTTCACCCTTTCACC
DR389 For1	GCCACCGCGCCCGGCCGATTATATACAAACCAACATTTCAG
DR389 Rev	AATTAAAATATTGCTGCCAAACG
qPCR	
FI-18a For	TCAGAAATCTTAGGTCATCGTATC
FI-18a Rev	TCTTGTTAATGGGCTTGTGG
FI-14a For	AAACTTCTGTTTGAGAGAATACG
FI-14a Rev	ATTTGCCTTTGATTTGTTGGT
FI-11a For	TTTGGAGGTAGTGTATTTTTCTTA
FI-11a Rev	CTTCCATAGCACATCATAGTTCA
DR259 For2*	GTTGGTTTGTCAGATTTGTGC
DR240 For2*	GTGGCAGACGGGGATTTG
DR389 For2*	GATCAGCGTATGGCGACTC
DR418 For2*	CTGATTGATACTTTCGTTCCTTG
SI-1 For	CGGCCTGGTCTAGGGTGTAT
SI-1 Rev	TGTTCTCGTCTAGTTTTAGTATC
SI-13 For	CGGCCACCAATGGACTCTTG
SI-13 Rev	TGCTGTGATAACCTCTAGGCT
SI-17 For	CGGCCCAAAAGTCTTTATCTTA
SI-17 Rev	GAGGCAGCAAACAAACACGAG
SI-3 For	CGGCCCAGAGATTCTGTTAC
SI-3 Rev	GACCACAGTGCAATCAAATTAG

PCR validation (5'-Flanks)	
N1 For1	CACAGTTCTGCCATCCATTTCTA
N2 For1	CATTTGGAGGGAAAGTTGGATTA
N3 For1	GAAAAATCAGTGGGGGAGAAAATC
N4 For1	CAATGGGCTCAACTGATGGA
N5 For1	GGGGCTCATGTTCAAAGTCC
N6 For1	CTCTTGAACTTTAAAAATAAGTTGGT
N7 For1	ATCAAAGAAATCTTGATTAAATGG
N8 For1	CCCTGAGTCCCTCCCTTTC
N9 For1	CTGAGAGAGCAGCGAAATAATGT
N10 For1	CTGAGCATATTCTTGCTGTCCAT
N11 For1	AGGTATTTTCTGGGCTTTCTCTG
N12 For1	ACTATAATAAAACAATTCTTCTTCAAA
N1 For2	GGTGGGAAAGAAAAATTAGGATGC
N2 For2	CTCTGAAGGAAGGTCAATGCTA
N3 For2	CCAAAGTGCTGAAGACAATAGAG
N4 For2	GGCTTCAATTCTGCAAACTCTACT
N5 For2	CCAGGAAAAGTAGAGGAAGTTAGG
N6 For2	ATCCAACCTCTTTCAGTTCTACC
N7 For2	GAACAGAAAAATAAGTATGTTGGC
N8 For2	TGTCAAGAGATATTTGTGCGTCC
N9 For2	GTAACTAATGTGGTAACTAACAGGA
N10 For2	GCTGGGGAAATACAAAGAGAG
N11 For2	TAAGAACAGGTGAACAGAGGGG
N12 For2	CCATACCCAGATACAAACCTTCA
PCR validation (3'-Flanks)	
AY102	GAGACCATCCCGGCTAAAAC
AY237	CTTGCAGTGAGCCGAGATCC
N1_3 For1	TTGACAGAATTTGGACAGAGCC
N2_3 For1	TAGGAGGCGTGGTAGGTGCG
N3_3 For1	TTCCTTTGTGGGGCACTAGA
N4_3 For1	TGCTAATAACTGCATCTACACCTC
N5_3 For1	AGGATTAGCTTCTGCCATTCTG
N6_3 For1	GTAGTTGTACCCCCTCTTCCTC
N7_3 For1	GCATGTATTAAGTTCAGGAGGTG
N8_3 For1	GCCTTCCTCTACATGCATTTTCTAT
N9_3 For1	AATTCAAAAATGCCAGCAGC
N10_3 For1	GATCCTAGGTCAGCAAAACATT
N11_3 For1	AGGGTGGAAATAATTACACTTTGAT
N12_3 For1	CTTTATCTGTGCCCCTTACCTG
N1_3 For2	AGCCGTCGGTGTGAAAATAGC

N2_3 For2	AAGGTAGCCAACCGTGGAAT
N3_3 For2	AATTTCTTTCTAGCTCTCATATGTTTA
N4_3 For2	AGTTTGCAGCATTGAGAGGA
N5_3 For2	CTGCTCTTCCTGCTTGTGTTAG
N6_3 For2	TTTAGTTATTGTTACCTTCATCATCA
N7_3 For2	CACTGAAAGCTGGGTAGGAAGAT
N8_3 For2	ATTTTCTATGAAGTTTAAGCATACAGTT
N9_3 For2	CACCCATTTTGAACACTTTGAA
N10_3 For2	CACTGTCTCAGGCATGTATAGC
N11_3 For2	CTACATACTGTTACCTTGGAGCATT
N12_3 For2	TGATTTATGTCTTCTTATGTTAGCAG

* were used with Rev primer from spike-in preparation
