# Supplementary material for: An advanced enrichment method for rare somatic retroelement insertions sequencing
Source: Mob DNA. 2018 Oct 31;9:31. doi: 10.1186/s13100-018-0136-1 (PMC6208084; doi:10.1186/s13100-018-0136-1)
Supplement: Supplementary file 3 — This file contains a table with result of qPCR analysis of selected insertions and spike-in controls. (PDF 37 kb) [file 13100_2018_136_MOESM3_ESM.pdf]

qPCR analysis of selected insertions and spike-in controls

Locus	Unnorm*	Norm1*	Norm2*
FI-11a	10.5 (0.14)	14.0 (0.22)	15.5 (0.02)
FI-14a	11.7 (0.21)	13.9 (0.22)	14.8 (0.12)
FI-18a	11.0 (0.06)	15.8 (0.19)	15.6 (0.06)
DR240	28.6 (0.14)	23.7 (0.29)	21.1 (0.07)
DR389	26.2 (0.10)	23.3 (0.32)	21.4 (0.04)
DR418	32.9 (0.73)	25.8 (0.14)	24.9 (0.09)
DR259	35.2 (0.2)	27.0 (0.44)	23.8 (0.02)
SI-1	22.2 (0.11)	19.9 (0.06)	21.4 (0.06)
SI-3	21.3 (0.04)	18.9 (0.08)	17.5 (0.05)
SI-13	22.4 (0.18)	20.0 (0.07)	18.8 (0.13)
SI-17	24.1 (0.19)	21.5 (0.13)	20.7 (0.12)

* - for each insertion Ct and standard deviation (in parentheses) is given
FI – fixed insertion present in each cell analyzed, DR – spike-in controls, SI – somatic insertions.
